# Gene Mutation Profiles in Primary Diffuse Large B Cell Lymphoma of Central Nervous System: Next Generation Sequencing Analyses

**DOI:** 10.3390/ijms17050683

**Published:** 2016-05-06

**Authors:** Milena Todorovic Balint, Jelena Jelicic, Biljana Mihaljevic, Jelena Kostic, Bojana Stanic, Bela Balint, Nadja Pejanovic, Bojana Lucic, Natasa Tosic, Irena Marjanovic, Maja Stojiljkovic, Teodora Karan-Djurasevic, Ognjen Perisic, Goran Rakocevic, Milos Popovic, Sava Raicevic, Jelena Bila, Darko Antic, Bosko Andjelic, Sonja Pavlovic

**Affiliations:** 1 Clinic for Hematology, Clinical Center of Serbia, Belgrade 11000, Serbia; bb.lena@gmail.com (M.T.B.); jecajelicic@yahoo.com (J.J.); zmzg@sezampro.rs (B.M.); idabakrac123@gmail.com (J.B.); milosmak13@gmail.com (D.A.); belab@imi.bg.ac.rs (B.A.); 2 Faculty of Medicine, University of Belgrade, Belgrade 11000, Serbia; 3 Institute of Molecular Genetics and Genetic Engineering, University of Belgrade, Belgrade 11010, Serbia; kostichelena@gmail.com (J.K.); bojanastanic@yahoo.com (B.S.); nadjapejanovic@gmail.com (N.P.); lucic.bojana@gmail.com (B.L.); nmtosic@sezampro.rs (N.T.); irenas24@gmail.com (I.M.); maja.stojiljkovic@yahoo.com (M.S.); dora_karan@yahoo.com (T.K.-D.); 4 Institute of Transfusiology and Hemobiology of Military Medical Academy, Belgrade 11000, Serbia; belabalint26@yahoo.com; 5 Seven Bridges Genomics, Belgrade 11000, Serbia; ognjen.perisic@gmail.com (O.P.); goran.rakocevic@sbgenomics.com (G.R.); milos.popovic@sbgenomics.com (M.P.); 6 Department of Histopathology, Clinical Center of Serbia, Belgrade 11000, Serbia; jjelicic86@gmail.com

**Keywords:** primary DLBCL CNS, *TP53*, *ATM*, *PTEN*, *SMO*

## Abstract

The existence of a potential primary central nervous system lymphoma-specific genomic signature that differs from the systemic form of diffuse large B cell lymphoma (DLBCL) has been suggested, but is still controversial. We investigated 19 patients with primary DLBCL of central nervous system (DLBCL CNS) using the TruSeq Amplicon Cancer Panel (TSACP) for 48 cancer-related genes. Next generation sequencing (NGS) analyses have revealed that over 80% of potentially protein-changing mutations were located in eight genes (*CTNNB1*, *PIK3CA*, *PTEN*, *ATM*, *KRAS*, *PTPN11*, *TP53* and *JAK3*), pointing to the potential role of these genes in lymphomagenesis. *TP53* was the only gene harboring mutations in all 19 patients. In addition, the presence of mutated *TP53* and *ATM* genes correlated with a higher total number of mutations in other analyzed genes. Furthermore, the presence of mutated *ATM* correlated with poorer event-free survival (EFS) (*p* = 0.036). The presence of the mutated *SMO* gene correlated with earlier disease relapse (*p* = 0.023), inferior event-free survival (*p* = 0.011) and overall survival (OS) (*p* = 0.017), while mutations in the *PTEN* gene were associated with inferior OS (*p* = 0.048). Our findings suggest that the *TP53* and *ATM* genes could be involved in the molecular pathophysiology of primary DLBCL CNS, whereas mutations in the *PTEN* and *SMO* genes could affect survival regardless of the initial treatment approach.

## 1. Introduction

Primary central nervous system (CNS) lymphoma represents a rare form of extranodal non-Hodgkin lymphoma (NHL), which accounts for approximately 3% of all intracranial neoplasia. The current WHO classification recognizes primary CNS lymphoma as a distinct histological subtype of lymphoma, with more than 95% of cases belonging to diffuse large B cell lymphoma of CNS (DLBCL CNS) [1]. Expression profiles of primary DLBCL CNS B lymphocytes, as well as the fact that they express mutated *IGHV-IGHD-IGHJ* rearrangements with biased usage of immunoglobulin heavy variable genes suggest the post-germinal center origin of these cells and antigen-dependent proliferation [2,3,4,5]. Hence, the expression of extracellular matrix and adhesion molecules, which enable the activated circulating B cells to localize in CNS, has also been investigated [6].

The pathogenesis of primary DLBCL CNS remains poorly understood due to the rarity of the disease, limited availability of biopsy-based tumor tissue and insufficient knowledge about the genomic basis of the disease. Advances in next-generation sequencing (NGS) technology allow for efficient and comprehensive analysis of the molecular genetic makeup of various solid tumors and hematologic malignancies, including primary DLBCL CNS [7,8,9,10,11]. By using the whole exome sequencing approach, recent studies have identified mutations in the genes involved in B cell proliferation and differentiation, B-cell receptor (BCR), Toll-like receptor (TLR) and NF-κB signaling pathway regulators, as well as genes involved in chromatin structure and modifications, cell cycle regulation and immune recognition [9,11,12]. Here, we have analyzed 19 cases of primary DLBCL CNS using the MiSeq system, the next generation sequencer with high accuracy, and the TruSeq Amplicon Cancer Panel (TSACP) for screening mutations in 48 solid cancer-related genes.

## 2. Results

### 2.1. Survival Data

Sixteen patients (84.2%) achieved overall treatment response (partial/complete remission), while three patients (15.8%) had initially chemotherapy-resistant disease with a lethal outcome within five months. Nine patients (56.2%), out of sixteen patients who achieved favorable treatment response, relapsed. On the close date in June 2015, 11 patients (57.9%) were still alive, with the median follow-up of 28 months. The median overall survival (OS) in our group of patients was 41 months (95% CI 25.43–56.57), while median event-free survival (EFS) was 37 months (95% CI 34.76–39.24). The patients with Eastern Cooperative Oncology Group (ECOG) performance status (PS) ˃2 had poorer EFS (log rank = 9.24, *p* = 0.026) and OS (log rank = 10.02, *p* = 0.018) compared to the patients with ECOG PS 0–2.

The following clinical variables did not reveal any impact on the EFS or OS when analyzed individually: age, presence of B symptoms and involvement of deep brain structures according to the Visco–Young algorithm (*p* > 0.05) [13]. However, the patients with biopsy or partial tumor resection had significantly shorter OS with the median of 28 months compared to the patients who had total tumor resection, whose median was not reached (log rank = 4.34, *p* = 0.037). Furthermore, EFS was longer in patients with total tumor resection in comparison to patients with partial tumor resection (median not reached *vs.* 24 months, log rank = 4.15, *p* = 0.042).

### 2.2. Mutational Profile of Primary DLBCL CNS Revealed by Targeted Next Generation Sequencing

To detect the mutational profile involved in the pathogenesis of primary DLBCL CNS, we analyzed an approximately 6.65 × 10^8^-bp sequence from 19 primary DLBCL CNS patients by targeted NGS using TSACP that covers 212 hotspot regions from 48 genes. TSACP comprises oncogenes and tumor suppressor genes involved in cell proliferation, apoptosis, genome stability and chromatin regulation. The average coverage of high-quality sequences was 835× per amplicon. Eight genes (*MPL1*, *FGFR3*, *CDKN2A*, *NOTCH1*, *HRAS*, *STK11*, *GNA11* and *SRC*) were discarded due to insufficient coverage; therefore, a total of 187 amplicons from 40 genes was used for subsequent analysis. Variants were identified in relation to the reference genome by applying a Bayesian approach and compared to public genetic variation databases.

**Figure 1:**
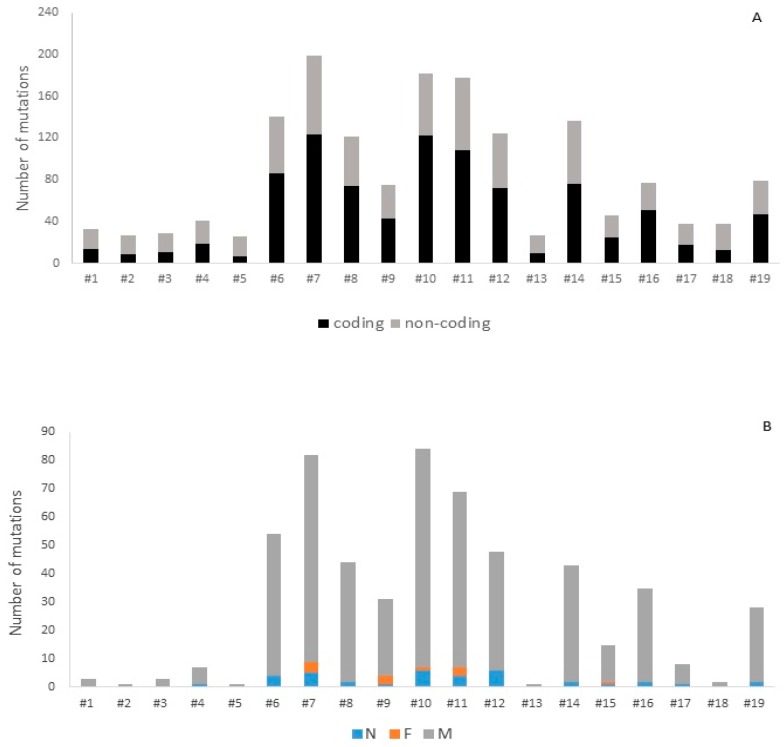
The number of mutations per primary DLBCL CNS patient. (**A**) Total number of mutations in coding and non-coding regions identified by targeted NGS; (**B**) distribution of nonsense (N), frameshift (F) and missense (M) mutations in coding regions of targeted genes.

The number of different variants detected in our study, in both coding and non-coding targeted regions, was 1247, out of which 825 variants were in the coding regions and 422 outside of the targeted exons. Among them, 38 were small indels, whereas 1209 were single nucleotide variants (SNVs). In 19 primary DLBCL CNS patients, we identified a total of 920 mutations in coding regions (median per patient: 43; range: 6–122) and 690 mutations in the non-coding regions (median per patient: 26; range: 17–76) (Figure 1A). Based on the biological significance of potentially protein-changing mutations, we focused on the genes containing nonsense (N), frameshift (F) and missense (M) alterations. Four patients had over 50 NFM mutations: #6, #7, #10 and #11 (Figure 1B). In all patients included in this study, a total of 559 mutations within coding regions (median per patient: 28; range: 1–84) were potentially protein-changing, including NFM mutations (Figure 1B).

In at least five out of 19 cases, we identified 28 genes containing potentially protein-changing mutations (Table 1).

**Figure 2:**
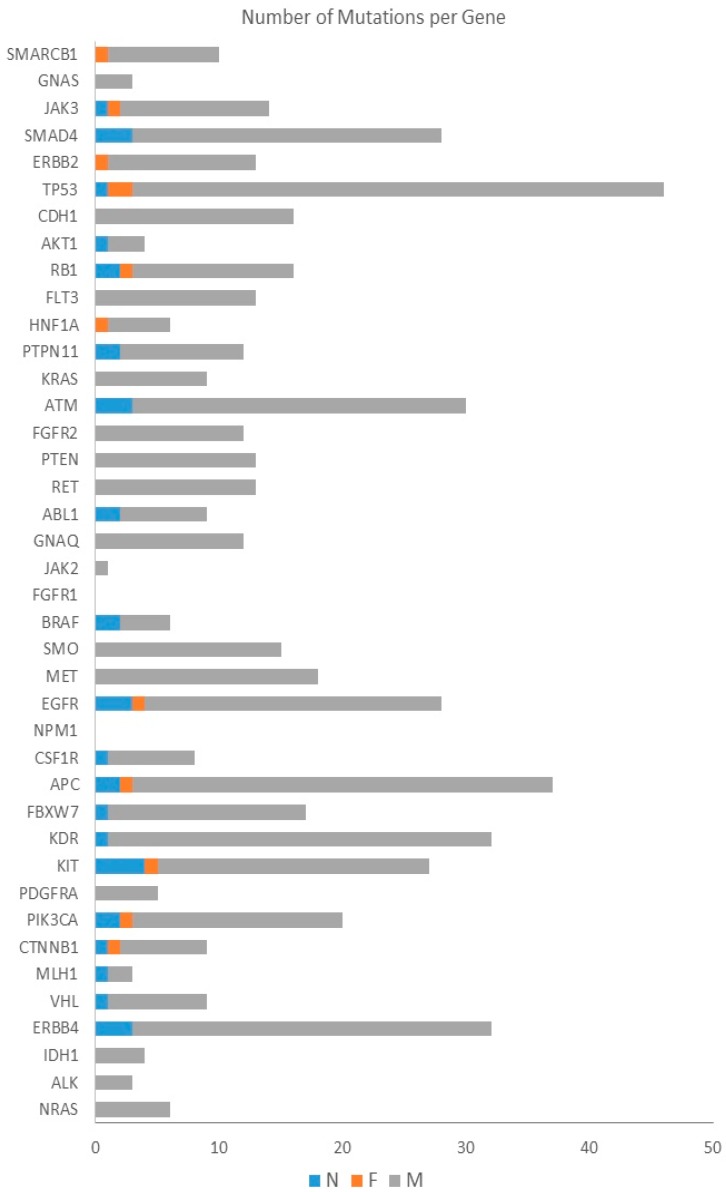
The number of mutations per targeted gene. Distribution of NFM mutations in the coding regions of targeted genes.

Some of the genes were highly mutated, harboring forty or more mutations (identified in the coding regions of seven targeted genes: *ERBB4*, *KIT*, *KDR*, *APC*, *EGFR*, *TP53* and *SMAD4*). On the other hand, targeted sequencing for the chosen panel of genes failed to detect any mutations in *NPM1* and *FGFR1*, while less than five mutations were identified in *MLH1*, *JAK2* and *GNAS* genes (Figure 2).

**Figure 3:**
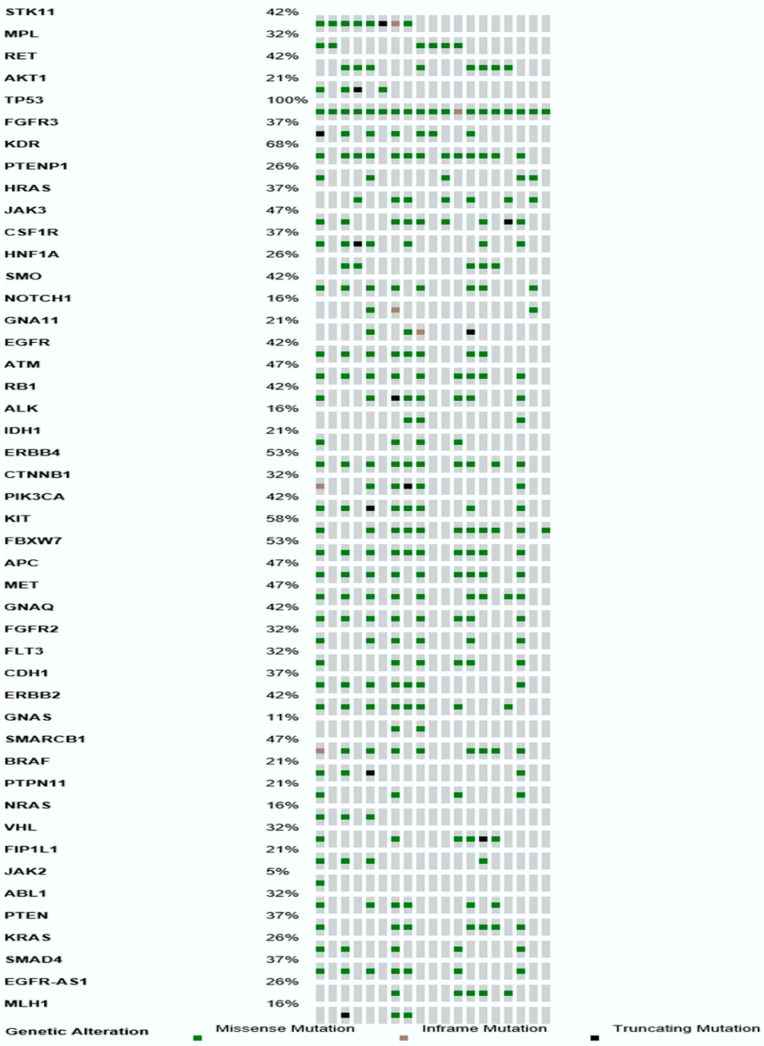
OncoPrint showing the distribution of genetic alterations in 40 targeted tumor suppressor and oncogenes in 19 primary DLBCL CNS patients. The type of mutations are labeled in the color legend, particular genes in rows and tumor samples in columns.

Over 80% of NFM mutations were detected in eight genes, *CTNNB1*, *PIK3CA*, *PTEN*, *ATM*, *KRAS*, *PTPN11*, *TP53* and *JAK3*, pointing to a potential role of these genes in lymphomagenesis. It is worth noting that 30 or more NFM mutations were detected in targeted sequences of five genes (*ERBB4*, *KDR*, *APC*, *ATM* and *TP53*). Furthermore, we found eight genes having at least one NFM mutation per patient: *ERBB4*, *PIK3CA*, *KIT*, *KDR*, *APC*, *EGFR*, *SMO*, *ATM* and *TP53* (Figure 2). The list of the genes and their mutation type are represented in Figure 3.

**Figure 4:**
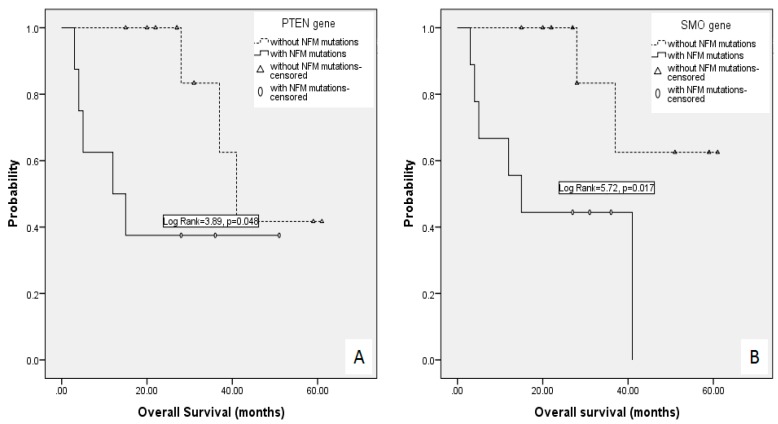
Overall survival according to the presence of NFM mutations in the *PTEN* gene (**A**) and the *SMO* gene (**B**).

Several genes appeared to be frequently affected by NFM mutations. Namely, *TP53*, *KDR*, *KIT*, *ERBB4* and *EGFR* genes were found to have more than 20 NFM mutations in over 50% of patients (10/19). Moreover, our findings reported 33 recurrent mutations that appear in at least two patients. All of the recurrent mutations are listed in Table S1. *TP53* was the only gene harboring mutations in all 19 primary DLBCL CNS patients, having on average over two NFM mutations per primary DLBCL CNS (range: 1–5). Our study has shown that NFM mutations in *TP53* were in correlation with the total number of mutations per primary DLBCL CNS patient. Genes *APC* and *ATM* were also shown to have a high mutation load of 37 and 30 mutations in nine patients, respectively. We have demonstrated a correlation between NFM mutations in *ATM* gene and the total number of NFM mutations per primary DLBCL CNS patient (*r* = 0.49, *p* = 0.032). In nine patients with a highly mutated *ATM* gene, we detected 1234 mutations (median per patient: 137; range: 75–199), and in the remaining 10 patients without any mutation in *ATM* gene, we identified 384 mutations (median per patient: 35.5; range: 26–79). Furthermore, the patients with NFM mutations in the *ATM* gene had inferior EFS (median 13 months *vs.* median not reached, log rank = 4.39, *p* = 0.036), but not OS (log rank = 3.21, *p* = 0.073), compared to the patients without these mutations in the *ATM* gene. NFM mutations in *PTEN* were associated with shorter OS (12 months *vs.* 41 months, log rank = 3.89, *p* = 0.048), but not EFS (log rank = 3.34, *p* = 0.068) (Figure 4A). Three out of four patients with mutations in the *PTEN* gene had the germinal center B cell (GCB)-type according to the Visco–Young algorithm. Mutations in the *SMO* gene correlated with earlier disease relapse (Fisher’s exact test *p* = 0.023). Moreover, the patients without NFM mutations in the *SMO* gene had superior OS (median not reached *vs.* 15 months, log rank = 5.72, *p* = 0.017) and EFS (median not reached *vs.* 13 months, log rank = 6.46, *p* = 0.011) (Figure 4B).

## 3. Discussion

Advances in NGS technology enable comprehensive exploration of somatic mutations in various solid tumors and hematologic malignancies, including primary DLBCL CNS [8,9,11]. The mutational landscape of primary DLBCL CNS has recently been investigated using the whole exome sequencing approach. Here, 19 primary DLBCL CNS patients have been analyzed, while most of the previous studies reported results with a lower number of patients [10,12,14].

While whole genome and exome sequencing represent comprehensive methods for the detection of germline mutations in cancers, these methods are constrained by low read depth, which limits their potential to detect SNVs with low allele frequencies (less than 5%), e.g., somatic mutations. On the other hand, targeted resequencing represents an alternative approach to the whole genome and exome sequencing, allowing for much higher read depths and greater accuracy in detecting low-frequency somatic variants [15,16]. Here, we used the TSACP, a previously-validated targeted gene panel, in order to detect somatic mutations in samples of 19 primary DLBCL CNS patients [17,18]. The overall coverage of the genes in the panel was satisfactory, and we suggest that this approach provided reliable results to understand the molecular basis of primary DLBCL CNS pathology. Eight genes had amplicons with significantly lower coverage (less than 100×) and, therefore, were excluded from our study. In accordance with the previously-published data, most of the low coverage amplicons had a GC content higher than 64%, suggesting that high GC% played a role in poor amplicon performance [19].

In one of the recent studies, Bruno *et al.* analyzed nine primary DLBCL CNS patients and identified recurrent somatic mutations in 37 genes involved in key biological processes, including transcription (*ETV6*, *IRF2BP2*, *EBF1*, *IRF4*, *TBL1XR1*), cell cycle (*PIM1*, *BTG1*), nucleosome assembly (*HIST1H1D*, *HIST1H2AC*) and cell adhesion (*MUC16*, *ACTG1*), as well as NF-κB, WNT and B-cell receptor signaling pathways [10]. Furthermore, Vater *et al.* have identified recurrently mutated genes involved in B cell proliferation and differentiation, TLR and NF-κB signaling pathway regulators, as well as genes involved in chromatin structure and modifications, cell cycle regulation and immune recognition [12]. Mutations in some of these genes (*MYD88*, *TBL1XR1*, *PIM1*, *CD79B*, *CARD11*) have previously been described in a number of studies using different methodologies, such as allele-specific PCR assays, Sanger sequencing and high-resolution SNP arrays [7,9,19,20,21]. Interestingly, two recent studies that used the whole exome sequencing strategy failed to identify somatic mutations in known cancer-associated genes [10,12]. On the other hand, a comprehensive study by Lawrence *et al.* proposed that extensive false positive findings that mask true driver mutations might be due to heterogeneity in the mutational processes in cancer [22]. According to the authors, large proteins (>4000 amino acids) are found to be mutated at a significant frequency, thus making some of them falsely associated with various cancers, e.g., MUC16 and PCLO in DLBCL [8,22,23]. After applying an integrated approach to accurately identify significantly mutated genes, Lawrence *et al.* have identified a short list of genes that have been previously reported to be associated with various types of cancers, such as *TP53*, *CDKN2A*, *PIK3CA*, *PTEN*, *ERBB1*, *KEAP1*, *NFE2L2*, *NOTCH1* and *FBXW7* [22]. Our study, using amplicon-based technology, demonstrated the presence of over 80% of NFM mutations detected in eight genes: *CTNNB1*, *PIK3CA*, *PTEN*, *ATM*, *KRAS*, *PTPN11*, *TP53* and *JAK3*. Moreover, *TP53*, *KDR*, *KIT*, *ERBB4* and *EGFR* genes were found to have more than 20 mutations in over 50% of primary DLBCL CNS patients. In the most recent work based on genome-wide analysis of 19 primary DLBCL CNS patients, novel recurrent alterations were detected, including *TOX* and *PRKCD*, that might help to differentiate primary DLBCL CNS from systemic DLBCL [13]. Our NGS analysis showed that primary DLBCL CNS shares certain gene mutations with other solid brain tumors, which could possibly explain the different clinical behavior of this type of lymphoma in comparison to the other types of aggressive lymphomas.

The *TP53* gene was the only one harboring mutations in all 19 primary DLBCL CNS patients, with an average of over two NFM mutations per patient. Previously-published studies pointed out that the mutations in *TP53*, the first identified tumor suppressor gene, were associated with various types of tumors, especially hematological tumors and DLBCL [24]. This study demonstrated that the presence of mutations in *ATM* was in correlation with higher total number of mutations (1234 in patients with mutated *ATM vs.* 384 in patients with unmutated *ATM*). Previous findings described the *ATM* gene as a crucial checkpoint kinase important for double-strand break repair and, therefore, the gene responsible for genome stability and integrity. Taken together, we focused on *ATM* and *TP53*, the genome stability and integrity guardian genes, considering the total number of mutations, the average number of mutations per patient and the number of patients containing the alterations in these genes. Furthermore, in our study, mutations in two genes that are frequently mutated in brain tumors, *PTEN* and *SMO*, were found to correlate with inferior OS, regardless of the initially applied treatment approach. The *PTEN* gene, encoding a phosphatase, modulates the cell cycle by preventing the entry of damaged cells in the cell cycle and, consequently, their rapid growth and division [25]. The mutations in *PTEN* have been associated with breast, prostate and thyroid cancer and melanomas, as well as brain tumors, such as astrocytoma, ependymoma and oligodendroglioma [25,26]. The available data suggest a decreased survival of glioma patients with mutated *PTEN*; however, there are no data in the literature evaluating the impact of *PTEN* mutations in patients with primary DLBCL CNS. Our NGS analysis has pointed out the potential role of the *PTEN* gene in the primary DLBCL CNS pathogenesis. Interestingly, three out of four GCB primary DLBCL CNS patients had NFM mutations in *PTEN*. Another gene, *SMO*, represents an oncogene that, if mutated, leads to increased susceptibility for developing malignant disorders. The mutations in *SMO* were firstly described in basal cell carcinoma and recently in brain tumors, meningioma and medulloblastoma [27,28]. Thus, it is not surprising that our results revealed a correlation between the presence of the mutated *SMO* gene and the earlier appearance of disease relapse and inferior EFS and OS.

## 4. Experimental Sections

### 4.1. Patients

In this study, we have analyzed 19 newly-diagnosed immunocompetent patients with primary DLBCL CNS who were treated at the Clinic for Hematology, Clinical Center of Serbia, from 2003–2013. The patients were initially evaluated according to the standard procedures using magnetic resonance imaging (MRI) or computed tomography (CT) in order to detect the CNS disease. The diagnosis was confirmed on the tumor tissue using standard histopathologic staining procedures. CT scans (thoracic and abdominal) and the bone marrow biopsy were performed in all patients. The patients and/or their families were informed about the forthcoming procedures and gave written approval before starting treatment. This study was approved by the Ethics Board of the Faculty of Medicine, University of Belgrade, No29/X-11.

All of the patient’s characteristics, treatment approaches and outcome are summarized and presented in Table 2. The median age at diagnosis was 54 years (range: 29–69 years) with a predomination of female gender (12, 63.2%). Regarding performance status (PS), 7 patients had Eastern Cooperative Oncology Group (ECOG) PS 1–2 (36.8%) and 12 patients had ECOG PS 3–4 (63.2%). Only 3 patients (15.8%) had B symptoms, while eight patients (42.1%) had an increased level of lactate dehydrogenase (LDH). Six patients (31.6%) had the involvement of deep locations within the brain (basal ganglia, corpus callosum, periventricular regions, brainstem and cerebellum). In order to define the cell of origin of the tumor, we have used the Visco–Young algorithm. This algorithm showed a concordance of over 92% with the molecular-based predictive model done by Rosenwald *et al*. in which the gene expression profile of the patients represents the predictor for survival [29]. According to the Visco–Young algorithm, 4 patients (21%) had the GCB-type of lymphoma, while the rest had the non-GCB type [13]. Regarding initial surgical treatment, total tumor resection was performed in 9 patients (47.4%), partial tumor resection in 7 patients (36.8%) and biopsy only in 3 patients (15.8%). After the initial treatment at the neurosurgery department and histopathological confirmation of primary DLBCL CNS, patients were treated with the De Angelis protocol [30]. Treatment response was evaluated using imaging techniques.

### 4.2. TruSeq Amplicon Cancer Panel Library Preparation and Sequencing

The TruSeq Amplicon Cancer Panel (TSACP) (Illumina Inc., San Diego, CA, USA) targets mutational hotspots in 48 cancer-related genes (*ABL1*, *AKT1*, *ALK*, *APC*, *ATM*, *BRAF*, *CDH1*, *CDKN2A*, *CSF1R*, *CTNNB1*, *EGFR*, *ERBB2*, *ERBB4*, *FBXW7*, *FGFR1*, *FGFR2*, *FGFR3*, *FLT3*, *GNA11*, *GNAQ*, *GNAS*, *HNF1A*, *HRAS*, *IDH1*, *JAK2*, *JAK3*, *KDR*, *KIT*, *KRAS*, *MET*, *MLH1*, *MLP*, *NOTCH1*, *NPM1*, *NRAS*, *PDGFRA*, *PIK3CA*, *PTEN*, *PTPN11*, *RB1*, *RET*, *SMAD4*, *SMARCB1*, *SMO*, *SRC*, *STK11*, *TP53* and *VHL*), selected in accordance with the nature of primary DLBCL CNS, since its behavior resembles a solid tumor. TSACP consists of 212 amplicons captured by pairs of oligonucleotides designed to hybridize flanking targeted regions of interest. Genomic DNA from 19 primary DLBCL CNS patients was isolated from formalin-fixed paraffin-embedded (FFPE) tumor tissue (Qiagen, Hilden, Germany). The library preparation was performed using 250 ng of genomic DNA, according to the manufacturer’s protocol. The sequencing was conducted on the MiSeq system (Illumina Inc., San Diego, CA, USA). Paired-end sequencing was performed using the MiSeq Reagent Kit v3 (600-cycle), and the sequencing quality was demonstrated by the percentage of bases having a Q30 score (1 error in 1000 bases) of 97.2%.

### 4.3. Bioinformatics Analysis

The first processing step was composed of the basic quality control performed with FastQC [31] and the trimming of low-quality bases (base quality <20) from read ends, which was performed with FastqMc [32]. The alignment to the GRCh37 reference genome, which produced BAM file(s), was done with BWA-MEM [33,34,35]. The indel realignment over the reads overlapping target regions was performed with the RealignerTargetCreator and IndelRealigner tools from GATK [36,37]. The additional quality control was done using custom scripts developed by SBG. The scripts counted reads in each amplicon and identified amplicons with systematically low read coverage across all samples. The variant calling and filtration were carried out with the GATK UnifiedGenotyper and VariantFiltration tools [36,37]. UnifiedGenotyper produced a VCF file containing single nucleotide variants (SNVs) and indel variants in relation to the GRCh37 reference genome by applying a Bayesian approach. As the final step, the VariantFiltration tool was used to filter out low-quality variants from the VCF file. Finally, a report was generated that summarizes per sample the results for all amplicons, including the sequence depth and, if present, the called mutation (both on the DNA and protein level) and the dbSNP identifier. The Integrated Genomics Viewer was used for visual evaluation of the data [38].

### 4.4. Statistical Analysis

In order to summarize patient’s characteristics, descriptive statistics calculated median values and ranges for continuous variables, while percentages and frequencies for categorical variables. Differences between groups were analyzed using Fisher’s exact test for categorical variables. The OS was defined as the time from the moment of diagnosis to either death or last follow-up. Event-free survival (EFS) was calculated as the time from the diagnosis to the date of either relapse or death. The Kaplan–Meyer method and the log rank test were used for survival analysis. All tests were two-tailed, and *p* ≤ 0.05 was considered as statistically significant. The IBM SPSS 21 (Chicago, IL, USA, 2012) package was used for these analyses.

## 5. Conclusions

Regarding the mutational status of the analyzed primary DLBCL CNS patients using NGS, we demonstrated that the *TP53* gene was mutated in all examined cases and that the patients with mutated *TP53* and *ATM* genes had a higher mutational load than other analyzed genes. Furthermore, *PTEN* and *SMO* emerged as the genes that correlated with the inferior overall survival of primary DLBCL CNS patients regardless of the initial treatment approach. Furthermore, mutations in *SMO* are associated with earlier disease relapse and shorter event-free survival.

## Figures and Tables

**Table 1:** Identification of 28 genes affected by potentially protein-changing mutations (nonsense, frameshift and missense) in at least 5 out of 19 primary DLBCL CNS patients.

Gene	Number of Mutations	Number of Patients
*TP53*	46	19
*KDR*	32	13
*KIT*	27	11
*ERBB4*	32	10
*EGFR*	28	10
*FBXW7*	17	10
*APC*	37	9
*ATM*	30	9
*MET*	18	9
*JAK3*	14	9
*RET*	13	9
*PIK3CA*	20	8
*RB1*	16	8
*ERBB2*	13	8
*GNAQ*	12	8
*SMAD4*	28	7
*CDH1*	16	7
*SMO*	15	7
*PTEN*	13	7
*SMARCB1*	10	7
*CSF1R*	8	7
*FLT3*	13	6
*FGFR2*	12	6
*VHL*	9	6
*CTNNB1*	9	6
*ABL1*	9	6
*KRAS*	9	5
*HNF1A*	6	5

**Table 2:** Clinical characteristics of 19 patients with diffuse large B cell lymphoma of central nervous system.

No.	Gender	Age >60 Years	ECOG PS	Tumor Localization	LDH	Visco–Young Algorithm	Treatment Approach **	Therapy Response	Disease Relapse	Vital Status
**1**	M	No	1	Superficial	Normal	non-GCB	TTR	PR	Yes	Alive
**2**	M	No	2	Superficial	Normal	non-GCB	TTR	CR	Yes	Alive
**3**	F	Yes	4	Superficial	Elevated	GCB	TTR	PR	No	Alive
**4**	M	No	3	Superficial	Normal	non-GCB	PTR	CR	Yes	Dead
**5**	F	No	2	Superficial	Normal	non-GCB	TTR	CR	No	Alive
**6**	F	No	3	Deep	N/A	non-GCB	TB	CR	Yes	Dead
**7**	M	Yes	4	Superficial	N/A	non-GCB	PTR	PD	Resistant disease	Dead
**8**	F	No	4	Deep	Normal	GCB	TTR	CR	No	Alive
**9**	M	No	3	Deep	Normal	non-GCB	TB	PR	No	Alive
**10**	F	No	3	Superficial	N/A	non-GCB	PTR	PR	Yes	Dead
**11**	F	Yes	1	Superficial	Normal	non-GCB	TB	PR	No	Alive
**12**	F	Yes	4	Deep	Normal	non-GCB	PTR	PR	Yes	Dead
**13**	F	Yes	3	Deep	Elevated	non-GCB	TTR	CR	Yes	Alive
**14**	F	Yes	4	Superficial	Elevated	GCB	TTR	PD	Resistant disease	Dead
**15**	F	No	4	Superficial	Normal	non-GCB	PTR	PD	Resistant disease	Dead
**16**	M	No	1	Superficial	Elevated	non-GCB	TTR	CR	No	Alive
**17**	M	No	4	Superficial	Elevated	non-GCB	PTR	PR	Yes	Dead
**18**	F	No	1	Superficial	Normal	non-GCB	TTR	CR	Yes	Alive
**19**	F	No	2	Deep	Elevated	GCB	PTR	PR	No	Alive

F, female; M, male; ECOG PS; Eastern Cooperative Oncology Group performance status; LDH, lactate dehydrogenase; GCB, germinal center B cell subtype; TTR, total tumor resection; PTR, partial tumor resection; TB, tumor biopsy; CR, complete remission; PR, partial remission; PD, progressive disease; N/A, not applicable. ** All patients received De Angelis chemotherapy after the initially surgical approach.

## Supplementary Materials

Supplementary materials can be found at http://www.mdpi.com/1422-0067/17/5/683/s1.
